# Supplementary material for: A Simple and Cost-Effective DNA Preparation Method Suitable for High-Throughput PCR Quantification of Hepatitis B Virus Genomes
Source: Viruses. 2020 Aug 24;12(9):928. doi: 10.3390/v12090928 (PMC7552047; doi:10.3390/v12090928)
Supplement: Supplementary file 1 [file viruses-12-00928-s001.zip › viruses-899755-supplementary.pdf]

A simple and cost-effective DNA-preparation method suitable for high-throughput PCR quantification of hepatitis B virus genomes

Eunji Jo[1], Jaewon Yang[1], Alexander Koenig[1], Seung Kew Yoon[2,3], and Marc P. Windisch[1,4*]

[1]Applied Molecular Virology Laboratory, Institut Pasteur Korea, 696 Sampyeong-dong, Bundang-gu, Seongnam-si, Gyeonggi-do, South Korea.
[2]Catholic University Liver Research Center, The Catholic University of Korea, Seoul, South Korea.
[3]Division of Gastroenterology and Hepatology, Department of Internal Medicine, College of Medicine, Seoul St. Mary's Hospital, The Catholic University of Korea, Seoul, South Korea.
[4]Division of Bio-Medical Science and Technology, University of Science and Technology, 217, Gajeong-ro, Yuseong-gu, Daejeon, South Korea.

*Correspondence: marc.windisch@ip-korea.org; Tel.: +82-31-8018-8180; Fax: +82-31-8018-8014

Supplementary Figures

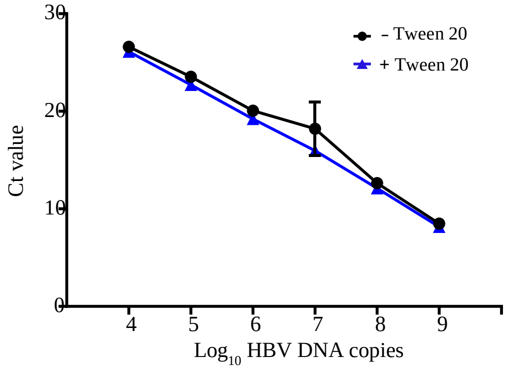

Figure S1. Effect of Tween-20 on qPCR. Standard curve of HBV plasmid DNA. DNA was quantified by qPCR analysis in the presence or absence of 5% Tween-20.

A

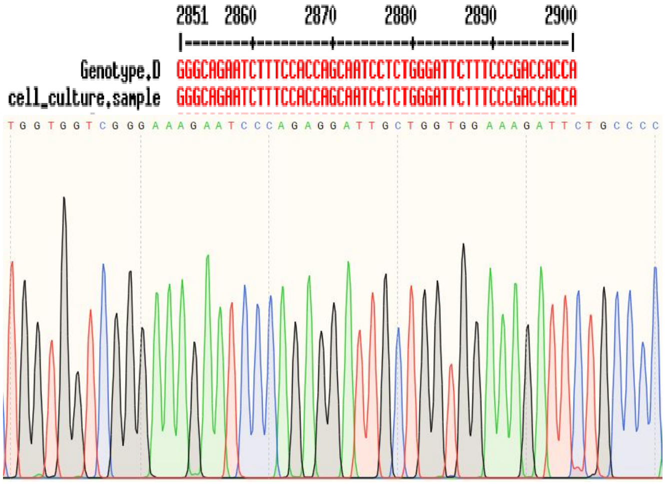

B

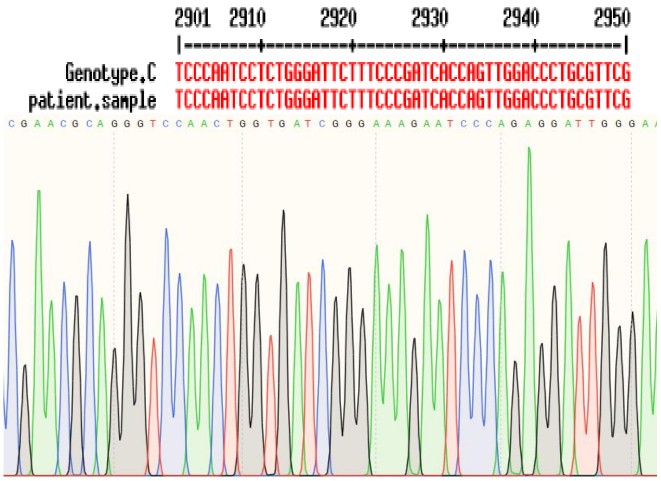

Figure S2. DNA sequencing of HBV DNA prepared using the column-free method. Chromatogram of DNA sequencing results from cell culture-derived HBV (A) and patient-derived HBV (B). HBV DNAs were isolated using a prep-free DNA preparation method, and the PreS1 through S genes were amplified using a Thermocycler Dice Touch (Takara Bio Inc., Shiga, Japan) with PrimeSTAR® HS Taq DNA polymerase (Takara Bio Inc., Shiga, Japan) as previously described [8]. Briefly, the pre-S1 through S regions were amplified using conserved universal primers (5'-TCACCATATTCTTGGGAACAAGA-3'and 5'-CGAAC CACTGAACAAATGGC-3'), and HBV genotypes were detected based on sequencing by Bionics (Seoul, Korea). An HBV-specific PreS primer [9] (5'-CCCTAGAAAATTGAGAGAAGTCCA-3') was used for sequencing. The sequences obtained were compared with published sequences covering the same genomic region of 8 HBV genotypes, as available in the National Center for Biotechnology Information GenBank.
